# Retention of laparoscopic psychomotor skills after a structured training program depends on the quality of the training and on the complexity of the task

**DOI:** 10.1007/s10397-016-0962-4

**Published:** 2016-07-16

**Authors:** Carlos Roger Molinas, Rudi Campo

**Affiliations:** 1 Neolife Medicina y Cirugía Reproductiva, Avenida Brasilia 760, 1434 Asunción, Paraguay; 2 European Academy of Gynaecological Surgery, Leuven, Belgium

**Keywords:** Laparoscopy, Training, Psychomotor, Intra-corporeal knot tying, Skills acquisition, Skills retention, LASTT model

## Abstract

This follow-up RCT was conducted to evaluate laparoscopic psychomotor skills retention after finishing a structured training program. In a first study, 80 gynecologists were randomly allocated to four groups to follow different training programs for hand-eye coordination (task 1) with the dominant hand (task 1-a) and the non-dominant hand (task 1-b) and laparoscopic intra-corporeal knot tying (task 2) in the Laparoscopic Skills Testing and Training (LASTT) model. First, baseline skills were tested (T1). Then, participants trained task 1 (G1: 1-a and 1-b, G2: 1-a only, G3 and G4: none) and then task 2 (all groups but G4). After training all groups were tested again to evaluate skills acquisition (T2). For this study, 2 years after a resting period, 73 participants were recruited and tested again to evaluate skills retention (T3). All groups had comparable skills at T1 for all tasks. At T2, G1, G2, and G3 improved their skills, but the level of improvement was different (G1 = G2 > G3 > G4 for task 1; G1 = G2 = G3 > G4 for task 2). At T3, all groups retained their task 1 skills at the same level than at T2. For task 2, however, a skill decay was already noticed for G2 and G3, being G1 the only group that retained their skills at the post-training level. Training improves laparoscopic skills, which can be retained over time depending on the comprehensiveness of the training program and on the complexity of the task. For high complexity tasks, full training is advisable for both skills acquisition and retention.

## Introduction

The ideal method for training in laparoscopic surgery is an issue of continuous debate and research. Although the classic apprentice-tutor model is still widely used, general agreement exists upon the importance of acquiring laparoscopic skills outside the operating room for ethical and practical reasons, such as the reduction of the operating time and the complications rates [1–5].

To facilitate the training and assessment of three specific basic laparoscopic psychomotor skills (i.e., camera navigation, hand-eye coordination, and bimanual coordination), the European Academy of Gynecological Surgery has developed an inanimate box model (i.e., the Laparoscopic Skills Testing and Training (LASTT) model) and demonstrated its feasibility, its face validity (the realism of the method), and its construct validity (the ability of the method to differentiate between novices and experts) [6, 7].

It has also been demonstrated in this model that training of basic laparoscopic psychomotor skills, specifically hand-eye coordination, facilitates the acquisition of more advanced skills, such as laparoscopic intra-corporeal knot tying [8]. Indeed, in contrast with trainees who did not follow the complete training program, trainees who trained hand-eye coordination with both the dominant hand (DH) and the non-dominant hand (NDH) registered a better starting level [8] and a shorten learning curve of laparoscopic intra-corporeal knot tying (unpublished observations).

In addition to laparoscopic psychomotor skills acquisition, the capacity to retain both basic and advanced skills is of outmost importance for defining an efficient laparoscopic training program. Some studies have already addressed this in different populations, using different models and scoring systems for both training and testing, and after different time points. The reported results are very consistent indicating that most skill remained better than at baseline. It is not sufficiently clear, however, the reasons why only some of them are retained at the post-training levels whereas some start deteriorating very soon [9–13].

This study was designed to evaluate the specific effect of different types of structured training programs upon laparoscopic psychomotor skills retention after a resting period of 2 years.

## Materials and methods

### Participants and venue

**Fig. 1:**
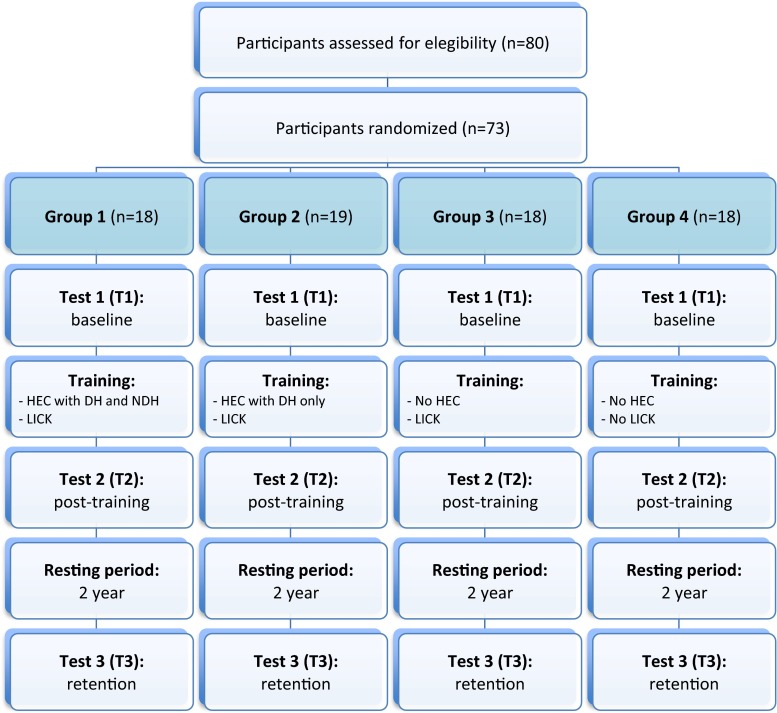
Flowchart of participation. *HEC* ham-eye coordination, *DH* dominant hand, *NDH* non-dominant hand, *LICK* laparoscopic intra-corporeal knot tying

The study was carried out in 2009 in the Centro Médico La Costa in Asunción, Paraguay, and intended to include the 60 gynecologists who had previously participated in a study aimed to evaluate laparoscopic skills acquisition, as reported previously [8], and the 20 gynecologists who were also recruited at that time specifically for the aims of this study on skills retention. These gynecologists had at that time sufficient experience in open and vaginal surgery but little or no experience in laparoscopic surgery (level 0–1 of the European Society of Gynecological Endoscopy classification) [6]. Participants who practice laparoscopic surgery or skills training between the previous and this study were excluded. A total of 73 participants were recruited for this study and their age, gender, training status (i.e., residents or specialists), and dominant hand side were recorded (Fig. 1). The remaining seven participants were not eligible for this study because they became experts in laparoscopy (*n* = 3) or were no longer accessible due to geographical limitations (*n* = 4).

### Instruments, materials, and tasks

The LASTT model, with the relevant materials for different tasks, was inserted into the Szabo trainer box (Karl Storz, Tutlingen, Germany). The tasks were performed with standard laparoscopic instruments (10 mm 0° optic, 5 mm Kelly dissection forceps, 5 mm Koh needle holders), and the optic was connected to an all-in-one (monitor, light source, and video camera) laparoscopic tower (Karl Storz, Tutlingen, Germany).

#### Task 1 (hand-eye coordination)

Participants navigated a camera with a 0° optic and grasped and transported six objects to six targets as described previously [8]. Briefly, they stood behind the trainer box in the midline. The optic was introduced through a midline port and the Kelly forceps through a lower and lateral port, to the right or the left according to the hand being evaluated. The Kelly forceps was held with the hand being evaluated and the camera with the contralateral hand. Participants were allowed to start the task when the first target and tip of the Kelly forceps were shown on the screen (start time). The matched targets (10 × 1 mm nails) and objects (5 × 4 mm open cylinders) were identifiable by color. The first object was grasped and transported to its target. Only when they succeeded in introducing the first cylinder into the first nail were they allowed to continue with the others in a fixed order. The task was executed and scored with both the DH (task 1-a) the NDH (task 1-b). The time for each repetition was limited to 600 s. The task finished either when the last object was transported to its target or when the time limit expired.

#### Task 2 (laparoscopic intra-corporeal knot tying)

Participants performed an intra-corporeal knot tying as described previously [8]. Briefly, they stood to the left of the trainer box. A soft pad with two pre-mounted sutures (vicryl 2-0, 20 cm length), 1 cm between entry and exit sites, and tails equally distributed at both sites was fitted in the Szabo trainer box in a horizontal position. The optic was introduced through a midline port and the needle holders through lower and lateral ports. The camera was fixed at a distance that allowed visualization of the entire operating field, and the needle holders were held with the relevant hands. The tip of the thread was grasped with the left needle holder, and the thread was pulled through the pad, leaving a 2-cm tail on the opposite side. Then, a double counter-clockwise knot was made, followed by a single clockwise knot and finally by a single counter-clockwise knot. The time for each repetition was limited to 600 s. The task finished either when the participant considered he/she completed the knot or when the time limit had expired. Then, the tutor performed a quality control, and only the flat and square knots were considered correctly performed.

### Scoring system

The measurement of the tasks was based on the time to correct performed exercise (TCPE), which reflects errors and economy of movements in the result and as such engages and accuracy assurance. Thus, when the task was successfully accomplished within the time limit, the score was the time actually used to execute the task, ranging from 1 to 600. However, if for any reason the task could not be successfully accomplished within the time limit, a penalty score of 1200 was established.

### Experimental design

At the time of the first study, 80 participants were randomly allocated to four different groups (G), according to the training program to be performed. Participants allocated to G4 were recruited specifically for the aims of this skills retention study, and therefore, they were disregarded for the first study about skills acquisition [8]. For the aims of the present study, 73 participants could be recruited again, remained all of them in the group originally assigned (Fig. 1).

To evaluate the baseline levels, all tasks were tested before training (T1) in sessions organized specifically for this aim. The test session started with detailed explanation and video demonstrations of the different tasks, and then, each participant performed three repetitions of task 1-a, task 1-b, and task 2. For each task, the average of the triplicate observations was used for statistical analysis [8].

Then, the assigned training program started. Training sessions of 1.5 h each were performed every 1 to 3 days, being approximately 1 month the average duration of the program [8]. In G1, the training program consisted in 60 repetitions of task 1-a and 60 repetitions of task 1-b in alternating order, followed by 60 repetitions of task 2. In G2, the training program consisted in 60 repetitions of task 1-a, followed by 60 repetitions of task 2. In G3, the training program consisted in 60 repetitions of task 2 directly, without any previous training for task 1. In G4, participants did not perform any training, not for task 1 nor for task 2.

To evaluate skills acquisition, the tasks were tested immediately after training (T2) in the same manner than for T1. The average duration in between T1 and T2 was 30 days.

To evaluate skills retention, after the sole effect of the exposition determined by this study, participants did not practice any type of laparoscopic procedure (no lab training nor surgery) and the tasks were tested again (T3) after a 2-year resting period in the same manner than for T1 and T2.

### Statistics

For evaluating the scores of all tasks (continuous variables), non-parametric tests were used because data were not normally distributed. Therefore, unless otherwise indicated, all data are presented as median (interquartile range). For G1, G2, and G3, scores before and after training were already reported in a previous study [8]. For statistical analysis of the present study, only the data of participants enrolled in both studies were included (i.e., data of participants of the previous study who did not participate in this study were excluded).

All statistical comparisons were performed using the GraphPad Prism Software, and two-tailed *P* values <0.05 were considered significant.

Intergroup differences at T1, T2, and T3 were evaluated with Kruskal-Wallis test and Dunn’s multiple comparison tests, whereas intra-group differences at the three time points were evaluated with Friedman test and Dunn’s multiple comparison tests. Intra-group differences between DH (task 1-a) and NDH (task 1-b) were evaluated with Wilcoxon test.

## Results

A total of 73 participants (G1 *n* = 18, G2 *n* = 19, G3 *n* = 18, G4 *n* = 18) were recruited (Fig. 1). All participants performed all assigned tasks, and their demographics (i.e., age, gender, training status, and DH side) were comparable and reported in Table 1.

**Table 1:** Participants’ demographics

	Groups			
	G1 (*n* = 18)	G2 (*n* = 19)	G3 (*n* = 18)	G4 (*n* = 18)
Age (median and range in years)	31 (28–47)	30 (28–39)	34 (29–47)	32 (28–45)
Gender (%)				
▪ Male	10 (55 %)	9 (50 %)	9 (50 %)	9 (50 %)
▪ Female	8 (45 %)	10 (50 %)	9 (50 %)	9 (50 %)
Training status (%)				
▪ Residents	8 (45 %)	11 (58 %)	8 (45 %)	7 (39 %)
▪ Specialists	10 (55 %)	8 (42 %)	10 (55 %)	11 (61 %)
Dominant hand side				
▪ Right	17 (94 %)	17 (89 %)	17 (94 %)	17 (94 %)
▪ Left	1 (6 %)	2 (11 %)	1 (6 %)	1 (6 %)

### Task 1-a (hand-eye coordination with the DH)

At T1, all groups had comparable scores, being 223 (174–279) for G1, 223 (112–350) for G2, 215 (162–266) for G3, and 194 (165–260) for G4 (all comparisons NS).

At T2, all groups that performed some kind of training improved their scores, being 44 (37–48) for G1 (*P* < 0.0001), 42 (37–51) for G2 (*P* < 0.0001), and 75 (62–95) for G3 (*P* < 0.0001), whereas G4 did not show any improvement and scored 201 (171–253) (NS). G1 scored similar than G2 (NS) and better than G3 (*P* = 0.001) and G4 (*P* < 0.0001). G2 scored better than G3 (*P* = 0.004) and G4 (*P* < 0.0001). G3 scored better than G4 (*P* = 0.03).

**Fig. 2:**
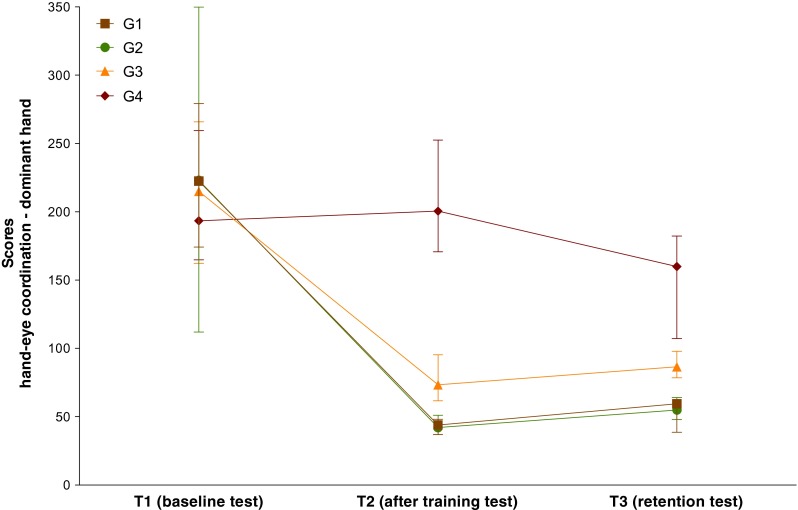
Skills for hand-eye coordination with the dominant hand (task 1-a). Participants were randomly allocated to different groups according to the training program (G1, G2, G3, and G4), and the skills were measured before training to evaluate the baseline levels (T1), immediately after training to evaluate skills acquisition (T2), and 2 years later to evaluate skills retention (T3). Median (interquartile range) scores are presented

At T3, all groups retained their skills almost at the same level than at T2, being 60 (39–63) for G1 (NS), 55 (48–64) for G2 (NS), and 87 (79–98) for G3 (NS), whereas G4 showed a slight improvement scoring 160 (107–182) (*P* = 0.02). Also, the intergroup differences detected at the end of the training program remains comparable at T3, G1 scoring similar than G2 (NS) and better than G3 (*P* = 0.001) and G4 (*P* < 0.0001) and G2 scoring better than G3 (*P* = 0.001) and G4 (*P* < 0.0001). However, due to the slight improvement in G4, differences between G3 and G4 were no longer significant (NS) (Fig. 2).

### Task 1-b (hand-eye coordination with the NDH)

At T1, all groups had comparable scores, being 342 (277–484) for G1, 343 (196–561) for G2, 358 (210–476) for G3, and 310 (205–730) for G4 (all comparisons NS).

At T2, all groups that performed some kind of training improved their scores, being 54 (45–63) for G1 (*P* < 0.0001), 71 (59–81) for G2 (*P* < 0.0001), and 92 (83–143) for G3 (*P* < 0.0001), whereas G4 did not show any improvement and scored 283 (202–364) (NS). G1 scored similar than G2 (NS) and better than G3 (P = 0.0001) and G4 (*P* < 0.0001). G2 scored similar than G3 (NS) and better than G4 (*P* < 0.0001). G3 scored better than G4 (*P* = 0.02).

**Fig. 3:**
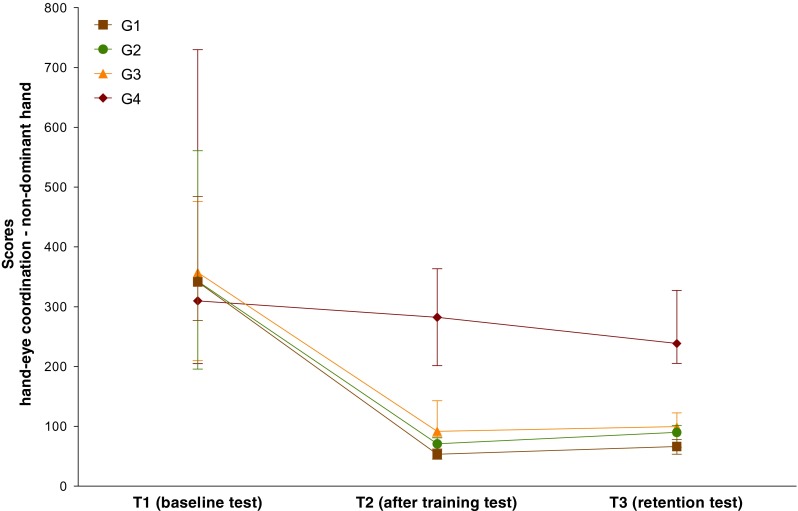
Skills for hand-eye coordination with the non-dominant hand (task 1-b). Participants were randomly allocated to different groups according to the training program (G1, G2, G3, and G4), and the skills were measured before training to evaluate the baseline levels (T1), immediately after training to evaluate skills acquisition (T2), and 2 years later to evaluate skills retention (T3). Median (interquartile range) scores are presented

At T3, all groups retained their skills at the same level than at T2, being 67 (54–78) for G1 (NS), 90 (65–102) for G2 (NS), 100 (95–123) for G3 (NS), and 239 (206–327) for G4 (NS). Also, the intergroup differences detected at the end of the training program remains comparable at T3, G1 scoring similar than G2 (NS) and better than G3 (*P* = 0.001) and G4 (*P* < 0.0001), G2 scoring similar than G3 (NS) and better than G4 (*P* < 0.0001), and G3 scoring better than G4 (*P* = 0.005) (Fig. 3).

### DH vs. NDH for hand-eye coordination

At all times points, all groups scored better for the DH than for the NDH (G1 *P* = 0.0001, *P* = 0.0003, *P* < 0.0001; G2 *P* = 0.01, *P* < 0.0001, *P* < 0.0001; G3 *P* = 0.0003, *P* < 0.0001, *P* < 0.0001; and G4 *P* < 0.0001, *P* < 0.0001, *P* < 0.0001, at T1, T2, and T3, respectively).

### Task 2 (laparoscopic intra-corporeal knot tying)

At T1, all groups had comparable scores, being 334 (231–1200) for G1, 250 (208–1200) for G2, 326 (202–1200) for G3, and 477 (341–1200) for G4 (all comparisons NS).

At T2, all groups that performed some kind of training improved their scores, being 32 (26–37) for G1 (*P* < 0.0001), 32 (28–44) for G2 (*P* < 0.0001), and 35 (31–37) for G3 (*P* < 0.0001), whereas G4 did not show any improvement and scored 459 (425–749) (NS). G1, G2, and G3 had comparable scores (NS), scoring the three groups better than G4 (*P* < 0.0001, *P* < 0.0001, *P* < 0.0001).

**Fig. 4:**
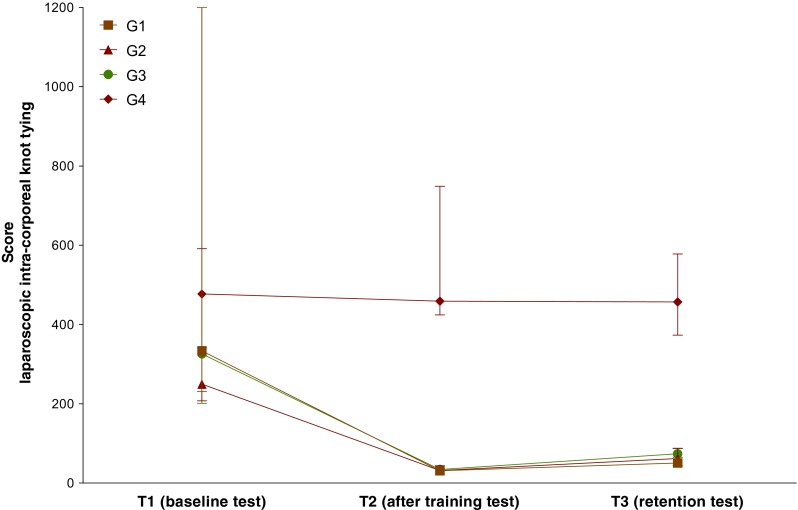
Skills for laparoscopic intra-corporeal knot tying (task 2). Participants were randomly allocated to different groups according to the training program (G1, G2, G3, and G4), and the skills were measured before training to evaluate the baseline levels (T1), immediately after training to evaluate skills acquisition (T2), and 2 years later to evaluate skills retention (T3). Median (interquartile range) scores are presented

At T3, G1 scored 51 (40–65), retaining the skills at the same level than at T2 (NS). G2 scored 62 (44–88) and G3 scored 74 (64–88), both groups scoring slightly worse than at T2 (*P* = 0.01, *P* = 0.008). G4 scored 457 (374–578) without any improvement in comparison with T2 (NS). Also, the intergroup differences detected at the end of the training program remains comparable at T3, having G1, G2, and G3 similar scores (NS), all of them better than G4 (*P* < 0.0001, *P* < 0.0001, *P* = 0.0005) (Fig. 4).

## Discussion

The general aim of this study was to evaluate laparoscopic skills retention after a structured training program. The specific objectives were to assess whether skill retention varies according to the training program and the laparoscopic task complexity. To evaluate the former, we used four different training programs and hypothesized that the program assigned to G1 would determine better results, whereas the program assigned to G4 would determine poorer results. To evaluate the latter, we used basic and advanced tasks and hypothesized that hand-eye coordination with the DH would be the easier to retain, whereas intra-corporeal knot tying would be the more difficult to retain.

Our data demonstrate that after a 2-year resting period, and without practicing any laparoscopic surgery o skill training, participants retained to a large extent the skills registered at the end of the training programs and that although some skill decay was noticed, they remained significantly better than at baseline. The level of retention, however, varied according to the group and the task analyzed, confirming our hypothesis.

Indeed, G1 had the better improvement after training (T2 vs. T1) and was able to retain the skills of the three tasks at the same level than 2 years earlier (T3 vs. T2), indicating the relevance of a comprehensive training for skills acquisition and retention. G4, however, did not show any improvement after training (T2 vs. T1), maintaining the sub-optimal skills execution in this study (T3 vs. T2) for more difficult tasks. For easiest task, however, a slight but significant improvement was observed, indicating a learning effect after few repetitions. G2 and G3 were in between the previous groups and had a significant improvement after training (T2 vs. T1). This was observed for trained and non-trained tasks, suggesting that intra-corporeal knot tying training may compensate to some extent the lack of hand-eye coordination training. Interestingly, both G2 and G3 were able to retain the hand-eye coordination skills, but not the intra-corporeal knot tying skills, at the same level than 2 years earlier, suggesting that retention of most difficult tasks may require more training.

Our data are consistent with the reports of other studies, but we must be cautious for general conclusions because the studies evaluating skills retentions differ significantly in study populations, training and tests programs/models, resting period, scoring systems, coaching and feedback, etc., as discussed in detail below.

Akdemir et al. evaluated skills retention in 11 first-year gynecology residents (without experience in laparoscopy) 6 months after a training program [9]. Although a matched control group was included for skills acquisition evaluation, there was no control group for skills retention evaluation. The 5-week program consisted in lectures (week 1) and 1-h practice session per week on a box trainer (weeks 2–5). The tasks performed in this box were well described, but the number of repetitions and the level of proficiency acquired were not reported. The baseline, post-training, and retention tests were done in another model (salpingectomy on the LapSim), and time, economy (path lengths and angular path), and error (blood loss and ovarian damage) were measured. The study concluded that the skills suffered a slight (angular path) or significant (time and path length) deterioration although they remained significantly better than at baseline [9].

Bonrath et al. evaluated skills retention in 36 medical students (without experience in laparoscopy) 6 or 11 weeks after a training program [10]. The 5-day program consisted in tutorials (day 1), baseline test (day 2), training (days 3–4), and post-training test (day 5). Participants, working in fixed pairs and accompanied by an expert for individual coaching, performed 4 cycles of nine tasks (navigation, grasping, transfer, positioning, cutting, loop tie, extra-corporal and intra-corporal knot tying and clipping) in a box trainer. The skills retention was evaluated after different time points (6 and 11 weeks) but unfortunately in two different groups. For testing purposes, time and errors of the same tasks performed during training were scored. The study concluded that skills are retained for at least 6 weeks and that deterioration, especially of the difficult tasks, started around 11 weeks [10].

Magaard et al. evaluated skills retention in a cohort of novices (*n* = 9) and experts (*n* = 10) 6 and 18 months after a training program [11]. The program (salpingectomy on the LapSim) consisted in 10 sessions (sessions 1–3: familiarization with the simulator, session 4: baseline test, sessions 5–9: training, session 10: after training test). The retention skills tests were done in the same model and time; economy and error (“bleeding”) were measured. The novices were tested after 6 and 18 months, and the experts only after 6 months. The study reported different performance for novices and experts. For novices, the skills were retained after 6 months, but they return back to the pre-training level after 18 months [11]. For experts, it seemed that the training program had no effect at all since they showed a constant performance from the baseline test up to the retention test at 6 months.

Hiemstra et al. evaluated skills retention in seven novices 1 year after a training program [12]. The seven-session program consisted in a baseline test (session 1), once a week training (sessions 2–6), and a final test (session 7). Participants performed five tasks on a box trainer (pipe cleaner, placing rubber band, placing beads, cutting circle, and intra-corporeal knot tying). Scores for speed and precision were measured. The retention test was performed in the same model. The study concluded that most basic skills acquired during a short training program sustain over time and that although some showed deterioration after 1 year, all skills remained better than before training [12].

De Win et al. evaluated skills retention in 145 medical students 1 and 6 months after different training programs [13]. Participants were randomly allocated to different groups according to training frequency (three sessions daily, two sessions daily, one session daily, one session on alternative days, one session per week, one session per week with optional additional practice in between sessions). All groups underwent six training sessions of 1.5 h each, consisting in three basic tasks (thumbnail, paperclip, needle rotation) to learn intra-corporeal suturing (session 1), needle positioning and penetration (session 2), and suturing (sessions 3–6). For the post-training and the retention tests, a 5-cm chicken-skin incision model was used and time was scored. The study concluded that once daily, 1.5-h session seems most beneficial for acquiring and for retaining intra-corporeal endoscopic suturing [13].

To a certain extent, our study counteract the limitations of these studies and extent their observations in several aspects. First, we enrolled a larger study population than most other studies. Second, our study comprises a longer resting period and ascertains that participants did not practice laparoscopic surgery or skill training during the 2-year resting period. Third, our study evaluates and discriminates between tasks of different levels of difficulties. Fourth, our study ascertains that during the skills acquisition phase, training was long enough to reach the plateau of the learning curve, in contrast with other studies in which participants were just briefly exposed to a task or allowed to practice it for a short period.

To be able to accomplish our objectives, the major challenge was to recruit the same participants of a study carried out 2-year earlier to evaluate skills acquisition. At that time, we recruited gynecologists with sufficient experience in open and vaginal surgery but without experience in laparoscopy. In order to avoid any confounding effect of additional training in laparoscopy, only those who did not performed any laparoscopic procedure during the resting period were included in the present study. This challenge was achieved because we were able to recruit 91.25 % of the participants of the previous study (90 % in G1, G3, and G4 and 95 % in G2), who were obviously 2 years older and some of them with other training status (many residents became specialists). Today, when gynecological laparoscopic surgery became a routine procedure in daily practice, this study population is no longer available worldwide. In our setting, however, this was still not the case; residents and specialists being exposed only to the tools and experience offered by the study design and hence being them a unique population for studying a variety of parameters that can affect training in laparoscopy. We are fully aware that this situation is difficult to reproduce and that might not be clinically realistic, because in most scenarios, an intensive 1-month training will not be followed by a 2-year period without exposure to laparoscopic surgery. On the contrary, one would be encouraged to apply one’s surgical training in the operating room to solidify hand memory and to advance one’s skills.

In conclusion, our study demonstrates that when using the European Academy of Gynecological Surgery inanimate box model, LASTT, laparoscopic skills retention depends on the quality of the previous training program and on the task complexity, suggesting that a comprehensive training is advisable to acquire and to retain laparoscopic skills. This indicates that laparoscopic psychomotor skills are comparable to swimming or cycling in the sense that they can be retained for longer periods of time once achieved proficiency. It remains unclear, however, the ideal method for a faster skills acquisition, which will require the evaluation of the characteristics of the learning curves of both basic and advanced laparoscopic skills, as well as the evaluation of other potential influencing factors, such as tutor feedback [14] and the additive effect of training others basic skills (e.g., camera navigation, bimanual coordination). And more importantly, it also remains to be demonstrated the predictive validity of these training models.
